# An Interdisciplinary Pain Rehabilitation Program for Veterans with Chronic Pain: Description and Initial Evaluation of Outcomes

**DOI:** 10.1155/2018/3941682

**Published:** 2018-04-17

**Authors:** Nidhi S. Anamkath, Sarah A. Palyo, Sara C. Jacobs, Alain Lartigue, Kathryn Schopmeyer, Irina A. Strigo

**Affiliations:** ^1^Department of Veterans Affairs, San Francisco VA Healthcare System, 4150 Clement Street, San Francisco, CA, USA; ^2^University of California, San Francisco, CA, USA

## Abstract

**Objective:**

Chronic pain conditions are prominent among Veterans. To leverage the biopsychosocial model of pain and comprehensively serve Veterans with chronic pain, the San Francisco Veterans Affairs Healthcare System has implemented the interdisciplinary pain rehabilitation program (IPRP). This study aims to (1) understand initial changes in treatment outcomes following IPRP, (2) investigate relationships between psychological factors and pain outcomes, and (3) explore whether changes in psychological factors predict changes in pain outcomes.

**Methods:**

A retrospective study evaluated relationships between clinical pain outcomes (pain intensity, pain disability, and opioid use) and psychological factors (depressive symptoms, catastrophizing, and “acceptable” level of pain) and changes in these outcomes following treatment. Multiple regression analysis explored whether changes in psychological variables significantly predicted changes in pain disability.

**Results:**

Catastrophizing and depressive symptoms were positively related to pain disability, while “acceptable” level of pain was idiosyncratically related to pain intensity. Pain disability and psychological variables showed significant changes in their expected directions. Regression analysis indicated that only changes in depressive symptoms significantly predicted changes in pain disability.

**Conclusion:**

Our results are consistent with evidence-based clinical practice guidelines for the management of chronic pain in Veterans. Further investigation of interdisciplinary treatment programs in Veterans is warranted.

## 1. Introduction

Chronic pain is a highly prevalent condition estimated to affect over 50% of Veterans who receive care through the Veterans Health Administration (VHA) [1, 2]. In addition, an investigation by Nahin [3] found that Veterans not only have high rates of pain but also generally report more pain than non-Veterans and have increased rates of severe pain. There is a large body of evidence showing that pain is associated with a plethora of deleterious consequences, including affective distress, long-term opioid use, greater utilization of healthcare services, and significant financial distress for patients and society [1, 4, 5]. More concerning is that the incidence of persistent pain in Veterans seems to be growing and standalone treatments, such as medication, physical therapy, or Cognitive-Behavioral Therapy may not be adequate for all patients [2, 5, 6]. To this end, the VHA has implemented a National Pain Management Strategy calling for integrated treatment to specifically improve pain management for Veterans nationwide [7, 8].

Pain is a multifaceted experience affected by genetic and biological vulnerabilities, as well as by psychosocial factors [9]. Thus, treatments that use a biopsychosocial model in the treatment of chronic pain have been recommended and have demonstrated good clinical outcomes [9–12]. Specifically, integrated biopsychosocial treatments are designed to facilitate functional restoration by addressing not only physiological processes of pain but also the cognitive appraisals and emotional reactions which may exacerbate the pain experience [13, 14]. One such psychological process known to affect the pain experience is pain catastrophizing, which is a multidimensional construct reflecting a collection of negative cognitions one has about experienced or anticipated pain [15]. Pain catastrophizing has shown to be a robust predictor of pain-related outcomes such as analgesic use and disability [15, 16]. Likewise, depression is another important psychological process, which has a dramatic negative effect on the pain experience [13, 17, 18]. A review of the comorbidity between depression and pain by Bair et al. [19] indicates that those with depression experience more intense pain for longer periods of time. Additionally, experimental investigations repeatedly show that individuals with depression have increased emotional reactivity to experimentally induced pain compared to never depressed controls [13, 17]. These results reinforce abnormal emotional processing in response to pain in those with depression, which therefore may exacerbate the pain experience for these individuals [20]. Of note, although often positively correlated, depression and catastrophizing have been shown to uniquely contribute to the pain experience, and thus, these two psychosocial facets should be examined separately [21–23]. Finally, one's perceived “acceptable” level of pain may also contribute to the pain experience. Clinically, “acceptable” level of pain is often assessed as a means of understanding patients' expectations for their pain care. Expectations regarding pain treatment and outcomes may drive motivations, adherence, and coping behaviors, thereby affecting overall treatment outcomes [24–26]. As such, it is crucial to understand how changes in one's expectation for what is an “acceptable level of pain” affect clinical pain outcomes.

The evidenced interplay between physiological, emotional, and cognitive aspects of pain warrants that these various components be addressed through treatment. Thus, the VHA has implemented interdisciplinary pain rehabilitation programs informed by the biopsychosocial model of pain to better address these different aspects of Veterans' pain experience [8]. Within the San Francisco Veterans Affairs Healthcare System (SFVAHCS), one such program, the Intensive Pain Rehabilitation Program (IPRP), was implemented in 2012 to address the increasing pain rehabilitation needs of Veterans. IPRP consists of several treatment components including the following: Acceptance and Commitment Therapy, Cogntive-Behavioral Therapy, physical therapy, pain education, and pharmacy counseling. One goal of the program has been to specifically address cognitive and emotional factors that may impact clinical pain outcomes such as pain intensity, pain-related disability, and current opioid medication use in Veterans with chronic, noncancer pain. Prior meta-analyses, which included studies mostly examining non-Veterans populations, have shown psychological interventions to be successful in reducing pain intensity, physical disability, and pain behaviors in individuals with chronic, musculoskeletal, noncancer pain [27, 28]. Several factors, such as decreased cognitive distortions and increased psychological flexibility, have been identified as potential mechanisms by which these treatment changes occur [29, 30]. However, there is a dearth of literature examining the relationship between cognitive and emotional psychological factors and clinical pain outcomes, particularly in Veterans receiving the highest, or “tertiary,” level of integrated pain rehabilitation. Such a treatment may be best suited to address the complex nature of the pain experience, especially in the veteran population, which has been shown to report more pain than the non-Veterans population [3]. Thus, to expand upon the current literature conducted primarily in the non-Veterans population, further investigation of treatment outcomes specifically in Veterans who attend an integrated pain rehabilitation program, such as IPRP, is warranted.

The present observational study had three goals. The first was to examine the relationship between clinical pain outcomes (pain intensity, pain-related disability, and opioid medication use) and cognitive and emotional psychological factors (subjective “acceptable” pain level, pain catastrophizing, and depressive symptoms) in Veterans undergoing a 12-week intensive interdisciplinary treatment at both baseline and follow-up. The second goal was to examine whether there were statistically significant changes in clinical pain outcomes, as well as in cognitive and emotional variables following treatment. The third goal was to explore changes in which psychological factors best predicted changes in clinical pain outcomes. An evolving understanding of the relationships between these factors in a veteran sample undergoing such integrated rehabilitation may further inform treatment development, assessments, and protocols.

## 2. Methods

Study procedures were approved by the SFVAHCS and University of California San Francisco Institutional Review Boards. Patient data were gathered from the Intensive Pain Rehabilitation Program (IPRP), which is an intensive and interdisciplinary treatment program designed for patients receiving care through the SFVAHCS who suffer from functionally impairing chronic, noncancer pain conditions. Inclusion in the program requires a referral from a clinician within the Veterans Affairs Healthcare System and an IPRP team-based evaluation to determine an individual's fit for the program. The patients in this sample attended the program three half-days a week for twelve weeks and were provided with education and self-management skills to facilitate practice at home. Skills were designed to help patients meet functional goals. All patients received the following components as part of the program: physical therapy (PT), CBT, Acceptance and Commitment Therapy (ACT), pain education (PEd), and pharmacy counseling (PharmC). PT included instructions on gentle movements, novel movement strategies, and self-applied massage techniques designed to increase body awareness, recover ease of movement, and re-engage in daily physical activities without causing a significant increase in pain. CBT focused on introduction of skills such as activity pacing, activity scheduling, relaxation training, and cognitive restructuring. Distraction was de-emphasized to be consistent with ACT, which focuses on participants developing mindfulness skills, being present-focused, and engaging in valued activities. PEd provided participants with education about chronic pain, including neurophysiology and neuroplasticity concepts, as well as healthy lifestyle choices as they relate to living with chronic pain. PharmC included education about the balancing risk and benefits of pain medications through group classes and individualized counseling with optimization of pain medication regimens when appropriate. Of note, opioid use reduction was not an explicit treatment goal of IPRP; however, IPRP supported the reduction of opioid medication as a patient-initiated goal or if a provider identified safety concerns.

As part of their clinical care, patients completed pre- and posttreatment measures. Data for this study were retrospectively examined from the clinical data collected by the program. Of the 55 patients who enrolled in the 3-day/week IPRP program between December 2012 and May 2015, individuals were excluded from data analysis if (1) they dropped out, (2) they completed the program but had not completed posttreatment questionnaires, (3) more than 15% of a questionnaire was missing either pre- or posttreatment (4) they were not Veterans, or (5) they were re-enrolled in the program (only the first enrollment was included in analyses to control for repetition effects). The final sample included 35 participants.

### 2.1. Measures

#### 2.1.1. Demographic and Clinical Data

Patients self-reported clinical and demographic information both pre- and posttreatment. Information included age, sex, race, years of education completed, duration of pain, and identified pain sites. Additionally, average or “usual” pain intensity (during the past week) and “acceptable” pain level were reported using a numeric rating scale (0–10). Of note, “acceptable” level of pain refers to an anticipated pain score with which a patient would be comfortable rather than acceptance of their pain condition. Here, it is a measure of patients' expectations regarding treatment.

#### 2.1.2. Pain Disability

The Pain Disability Questionnaire (PDQ) [31] is a 15-item measure of pain-related disability. Each item is rated from 0 to 10 with higher scores indicating greater pain-related disability. The measure is divided into two subscales. The first is the functional status component which reflects general functioning; the second is the psychosocial component. Scores on the PDQ are broken down to categorize pain disability as mild/moderate (0–70), severe (71–100), or extreme (101–150) [32].

#### 2.1.3. Pain Catastrophizing

The Pain Catastrophizing Scale (PCS) [15] is a 13-item self-report measure comprising three subscales used to assess an individual's negative cognitions (rumination, magnification, and hopelessness) about actual or anticipated pain. Items are rated using a 5-point Likert scale (0 = not at all and 4 = all the time). The PCS is a widely used measure of pain cognitions amongst a variety of chronic pain populations and has been shown to have good internal consistency [15, 33].

#### 2.1.4. Depression

The Patient Health Questionnaire (PHQ9) [34] is a nine-item self-report measure of depressive symptoms. Questions correspond to diagnostic criteria from the *Diagnostic and Statistical Manual of Mental Disorders*, 4th edition. Each item is rated from 0 to 3 (0 = not at all and 3 = nearly every day). Scores are broken down into five categories: minimal depression (0–4); mild depression (5–9); moderate depression (10–14); moderately severe depression (15–19); and severe depression (20–27). The questionnaire has been shown to be reliable and has good sensitivity and specificity [34, 35].

#### 2.1.5. Pharmacy Data

A pharmacist collected information on the number and type of medications that patients were taking specifically for their pain. Medication types included opioids, antidepressants, anticonvulsants, muscle relaxants, nonsteroidal anti-inflammatory drugs (NSAIDS), topical agents, and a category for other miscellaneous pain relievers. In addition, morphine daily equivalent doses (MEDD) were calculated for each patient as a measure of opioid medication use.

#### 2.1.6. Treatment Satisfaction

Five questions from the Pain Outcomes Questionnaire [36] which reflect treatment satisfaction (TxSat) were asked posttreatment. Each question asks participants to rate their satisfaction of the care they received on a scale from 0 to 10 (0 = no satisfaction and 10 = complete satisfaction). The questions ask patients to rate their satisfaction with the overall treatment, staff (personality and competence), and treatment schedule, as well as whether they would recommend the treatment. Rating of individual components of the program was not administered to this cohort but is being implemented currently.

### 2.2. Data Analysis

All statistical analyses were performed using IBM SPSS Statistics Version 24 (IBM, Chicago, IL). Parametric tests were used for all analyses. Demographic information and descriptive statistics for all outcome measures have been provided. Pretreatment correlations and posttreatment Pearson's correlations were investigated amongst variables (usual pain, acceptable pain, PDQ, PCS, PHQ9, MEDD, and TxSat). To assess differences in pre- and posttreatment scores for outcome variables, paired *t*-tests were performed. For all analyses, *p* < 0.05 was considered significant.

Lastly, due to the interdisciplinary nature of the IPRP which aimed to improve cognitive and emotional symptoms associated with chronic pain, we wanted to explore changes in what emotional and cognitive symptoms best predicted changes in pain outcomes in our study. Hence, a multiple linear regression model was conducted for pre-post changes in pain-related disability as a pain outcome and acceptable level of pain, PHQ9, and PCS as psychosocial predictors. Associations among individual demographic factors (age and sex) and outcomes were tested before running each regression to identify potential covariates. These predictors were chosen based on the components of the program that are meant to target cognitive and emotional symptoms of chronic pain.

## 3. Results

### 3.1. Veteran Sample Characteristics

Individuals in this sample were primarily male (71%) and Caucasian (62.9%). The mean age was 56.2 years (SD = 7.9). Means and standard deviations for pre- and posttreatment variables are presented in Table 1. As can be seen in Table 1, on average, patients' self-reported scores at baseline (or pretreatment self-reported scores) indicated moderate depression and extreme disability. Average pain duration (in years) and number of pain sites reported were 18.7 (SD = 13.2) and 7.9 (SD = 4.4), respectively. Table 1 also depicts sample characteristics for medication use, which showed that majority of these patients were receiving opioids.

### 3.2. Bivariate Associations

Pre- and posttreatment bivariate correlations are depicted in Tables 2 and 3, respectively. Usual pain was significantly correlated with the acceptable level of pain only at baseline (*r*=0.39,  *p* < 0.05) but not with PCS or PHQ at neither pre- nor posttreatment. PDQ was significantly correlated with PHQ (*r*=0.64,  *p* < 0.01) and PCS (*r*=0.51,  *p* < 0.01) but was not significantly related to the acceptable level pain. Posttreatment correlations between PDQ and PCS remained positive, but only the relationship to PDQ and PHQ remained significant (*r*=0.50,  *p* < 0.01). MEDD did not significantly relate to any of the psychological outcomes both pre- and posttreatment in our sample.

### 3.3. Comparison of Pre- and Posttreatment Outcomes

Paired *t*-tests for pre- and posttreatment values from completed self-report measures are presented in Table 4. Results indicate significant decreases in all variable scores except for “acceptable pain levels,” which significantly increased, as expected. Conversely, no significant changes were observed for pain intensity (usual pain) or for daily morphine equivalent dose (although tendencies were noted).

### 3.4. Exploratory Regression Analyses

The results of exploratory stepwise linear regression analysis with changes in pain disability (PDQ) as a dependent variable and changes in three predicting factors (depression, acceptable level of pain, and catastrophizing) are shown in Table 5. Improved pain disability scores (PDQ) were significantly predicted by improvements in depression scores, whereby changes in PHQ9 explained most of the changes in pain-related disability; adding acceptable levels of pain and/or catastrophizing did not improve the model.

## 4. Discussion

The present study retrospectively examines a sample of Veterans enrolled in interdisciplinary pain rehabilitation with the goal of exploring (1) the relationships between cognitive and emotional psychological variables and clinical pain outcomes, (2) changes in these psychological variables and outcomes following interdisciplinary treatment, and (3) whether changes in psychological variables predict changes in clinical pain outcomes. Expanding upon the existing literature, which has primarily evaluated interdisciplinary pain rehabilitation in the non-Veterans population, our findings indicate distinct relationships between pain-related clinical outcomes and the assessed psychological processes. Additionally, we found that the 12-week interdisciplinary pain rehabilitation program shows promise in improving pain-related psychological factors and pain-related disability in a mixed sample of extremely disabled, moderately depressed Veterans with severe chronic pain in multiple body sites. Lowering depressive symptoms may predict improvements in disability, and given the limited nonpharmacological options available to Veterans with such disability, these promising findings merit further examination.

We found several associations between clinical pain outcomes (i.e., self-reported pain intensity, pain-related disability, and opioid medication use) and the evaluated psychological measures, suggesting that pain-related clinical outcomes may be differentially influenced by the underlying cognitions and emotion. Specifically, pain intensity was significantly and positively associated with subjective acceptable pain level but not with pain catastrophizing or depressive symptom severity at both pre- and posttreatment. In contrast, pretreatment pain-related disability was positively related to depressive symptoms severity and pain catastrophizing but not with subjective acceptable level of pain. Such idiosyncratic relationships between the pain-related clinical outcomes and psychological features may have important implications for treatment planning based on a patient's specific goals. As an example, IPRP may choose to focus on functional restoration for a patient and use behavioral interventions (i.e., CBT and/or ACT) to target depressive symptom severity and pain catastrophizing, as these psychological processes were distinctly related to pain-related disability and therefore have the potential to impact pain-related debilitation if reduced. Targeting underlying processes that may affect the pain experience has indeed become a topic of interest in the literature [37], and further consideration of these distinctive relationships between pain outcomes and psychological processes has the potential to optimize treatment recommendations.

A comparison of pre- and posttreatment scores demonstrates that the administered intensive and interdisciplinary treatment significantly lessened negative cognitions and emotions associated with chronic pain, as well as subjects' perceived disability level, but had less effect on self-reported pain intensity and opioid use. Regarding the lack of improvement in pain intensity, this suggests that decreases in disability were not necessarily a function of decreases in pain intensity and is consistent with the extant literature and with the VHA National Pain Strategy [8], which calls for improvements in both physical and psychosocial functioning. Although decreases in pain intensity following interdisciplinary rehabilitation often occur, such reductions may not be necessary for improvements in functioning [38, 39]. This directly supports the foundational theory of the Acceptance and Commitment Therapy (ACT) treatment model, which hypothesizes that one's orientation to a distressing experience can be altered without an alteration in the distressing experience itself [29, 40]. Also, in line with the ACT model, decreases of negative psychological states, for example, depression and pain catastrophizing, may facilitate increases in behavioral flexibility. Increases in behavioral flexibility allow patients to re-engage in life activities reflecting personal values and thus decrease disability. Although behavioral flexibility was not measured in this initial sample of Veterans undergoing IPRP, it is plausible that our integrative treatment that improved depression and pain catastrophizing may have decreased disability by giving patients the option to engage in more value-based behaviors which they may have been avoiding previously [41, 42]. The significant impact that depression and catastrophizing may have on decreasing disability further supports the idea that decreases in pain intensity may not be needed for functional improvement.

Likewise, we found that opioid medication use did not show significant decrease although reduction tendencies were noted. Given the current state of the opioid epidemic [43, 44] and its relevance to the veteran population in particular [45–47], this observation warrants particular attention. Given that opioid dose reduction was not an explicit treatment goal of IPRP, lack of significant decrease in opioid medication use is not surprising. While there is evidence that similar interdisciplinary programs may be effective in reducing opioid intake, these programs often focus on opioid use reduction as part of their treatment or mandate cessation of opioids altogether [48, 49]. However, our findings are consistent with the existing literature [50] suggesting that decreases in opioid use may not be related to decreases in pain-related disability and/or improvements in psychological outcomes. This finding is promising as many patients who refuse to reduce opioid intake for fear of worsening of their pain symptoms may still be able to make functional gains. If such functional gains are made, patients may be more willing to initiate changes in their opioid use with the help of their treatment providers. Importantly, future follow-up investigations should examine whether participants require more time after treatment has completed to solidify newly acquired functional gains that would support greater reductions in opioids use.

Finally, exploratory regression analyses indicated that only changes in depressive symptoms severity significantly predicted changes in pain-related disability in the current sample. While other studies also found that changes in catastrophizing may also be predictive of improvements in pain disability, which we did not observe, our results lend further support to the strong relationship between depression and pain-related disability which has been noted in the existing literature [21, 51–53]. In line with the present finding, investigations have repeatedly found that depressive symptoms in patients with chronic pain are associated with increased levels of disability [19, 54]. Additionally, a longitudinal investigation of a non-Veterans sample by Scott et al. [53] found that reductions in depression significantly predicted both decreases in disability days and decreased likelihood of severe disability. Contrary to our findings, Scott et al. [53] found that levels of pain catastrophizing also predicted decreased levels of high disability and disability days, although the effect size was moderate compared to the effects of depression in their study. Thus, focusing on lowering depressive symptoms through value-based actions, cognitive restructuring, and other psychological techniques may be critical in increasing functional improvements in veteran population. Yet another study showed that that catastrophizing had a larger role in predicting disability levels than depression [21]. Although the findings regarding whether depression or catastrophizing is more important for pain-related disability are mixed, the literature is consistent in indicating that both psychological factors play an important role. Thus, future investigations of catastrophizing in a larger sample of Veterans in interdisciplinary treatment may indeed predict changes in disability.

Several limitations of the present study should be noted. Firstly, the current sample was restricted to those patients who had minimal missing data (<10%), which limited the research and associated conclusions in the following ways: (a) possible introduction of selection bias, (b) limited sample size, and (c) lack of posttreatment follow-up data. Such limitations indeed impact the generalizability of our findings, and further research with a larger and more inclusive sample is needed. Thus, our initial findings should be interpreted with caution, particularly regarding the nonsignificant reduction in average reported pain intensity. We are currently collecting data in a larger sample of Veterans and at multiple assessment time points. Additionally, a future examination with a larger and less restrictive sample would also allow for a more fine-grained analysis of the outcome variables and identification of potential mechanisms of change. Furthermore, all data collected were self-reported, and results are subject to possible over- and underreporting. Future examinations should include additional objective measures of functioning, such as physical therapy outcomes. Despite these limitations, these initial results are promising and clearly demonstrate the need for further evaluations in our veteran population. Specifically, due to apparent lack of comparative efficacy trials for nonpharmacological pain treatment options available to our Veterans, the investigation of the efficacy of such a program via a randomized controlled trial is desperately needed.

## 5. Conclusion

Chronic pain is a major concern among Veterans, leading to immense suffering and disability. Interdisciplinary treatment of chronic pain has been recommended by the VHA, and appraisal of such a nonpharmacological treatment in this specific population is needed. Preliminary evaluation of such a treatment program shows intensive and interdisciplinary pain rehabilitation to be a promising treatment for Veterans with chronic pain. Patients overall exhibited positive gains in cognitions and emotions related to their pain experience, as well as improved functioning. These improvements among this sample are promising and are a call to action to conduct efficacy trials going forward.

## Figures and Tables

**Table 1:** Veteran sample characteristics (*n*=35).

	Pretreatment	Posttreatment
Demographic		
Mean age in years (SD)	56.2 (7.9)	—
Male sex, *n* (%)	25 (71%)	—
Caucasian race, *n* (%)	22 (62.9%)	—
Mean pain duration (SD), *n*=33^#^	18.70 (13.20)	
Mean number of pain sites (SD)	7.89 (4.40)	6.94 (4.26)
Reported pain sites, *f* (%)		
Leg	26 (74.29%)	26 (74.29%)
Low back	32 (91.43%)	30 (85.71%)
Mid-back	22 (62.86%)	18 (51.43%)
Upper back	17 (48.57%)	12 (34.29%)
Head	12 (34.29%)	9 (25.71%)
Neck	25 (71.43%)	20 (57.14%)
Shoulder	19 (54.29%)	20 (57.14%)
Buttocks	19 (54.29%)	12 (34.29%)
Foot	15 (42.86%)	16 (45.71%)
Jaw	10 (28.57%)	7 (20.00%)
Chest	6 (17.14%)	3 (8.57%)
Abdomen	7 (20.00%)	4 (11.43%)
Arm/hand	17 (48.57%)	13 (37.14%)
Fingers	14 (40.00%)	10 (28.57%)
Toes	12 (34.29%)	7 (20.00%)
Face	4 (11.43%)	2 (5.71%)
Genitals	6 (17.14%)	5 (14.29%)
Others	8 (22.86%)	8 (22.86%)
Medications, *f* (%)		
Opioids	26 (74.29%)	25 (71.43%)
Antidepressants	16 (45.71%)	18 (51.43%)
Anticonvulsants	17 (48.57%)	17 (48.57%)
Muscle relaxants	14 (40.00%)	16 (45.71%)
NSAID	13 (37.14%)	15 (42.86%)
Topical agents	20 (57.14%)	22 (62.86%)
Others	8 (22.86%)	8 (22.86%)
Clinical pain outcomes, mean (SD)		
Usual pain^&^	6.40 (1.94)	5.76 (1.69), *n*=34^#^
PDQ	103.14 (23.01)	87.77 (24.40)
MEDD	69.68 (100.88), *n*=34^#^	62.32 (91.90), *n*=34^#^
Psychological outcomes, mean (SD)		
PHQ9	14.29 (6.23), *n*=34^#^	9.89 (5.50)
PCS	23.78 (12.21), *n*=32^#^	13.85 (8.46), *n*=33^#^
Acceptable pain^&^	2.63 (1.50), *n*=32^#^	3.76 (1.50), *n*=34^#^
Others		
Treatment satisfaction	—	46.06 (5.99)

SD = standard deviation; NSAID = nonsteroidal anti-inflammatory drugs; PHQ9 = Patient Health Questionnaire; PCS = Pain Catastrophizing Scale; PDQ = Pain Disability Questionnaire; MEDD = morphine equivalent daily dose; ^&^numeric rating scale from 0 (no pain) to 10 (worst pain imaginable). ^#^The number of patients is lower than the total number of patients (*n*=35).

**Table 2:** Pretreatment correlations.

Variable	1	2	3	4	5	6
(1) Usual pain^&^	—	0.39^∗^	0.20	0.08	0.39^∗^	−0.08
(2) Acceptable pain^&^		—	0.03	−0.07	0.26	−0.08
(3) PHQ			—	0.54^∗∗^	0.64^∗∗^	0.15
(4) PCS				—	0.51^∗∗^	0.10
(5) PDQ					—	0.25
(6) MEDD						—

PHQ9 = Patient Health Questionnaire; PCS = Pain Catastrophizing Scale; PDQ = Pain Disability Questionnaire; MEDD = morphine equivalent daily dose; ^&^numeric rating scale from 0 (no pain) to 10 (worst pain imaginable); ^∗^correlation is significant at the 0.05 level (2-tailed); ^∗∗^correlation is significant at the 0.01 level (2-tailed).

**Table 3:** Posttreatment correlations.

Variable	1	2	3	4	5	6
(1) Usual pain^&^	—	0.34	0.21	0.29	0.36^∗^	−0.17
(2) Acceptable pain^&^		—	−0.18	−0.14	0.01	0.05
(3) PHQ			—	0.45^∗∗^	0.50^∗∗^	0.31
(4) PCS				—	0.34	−0.08
(5) PDQ					—	0.20
(6) MEDD						—

PHQ9 = Patient Health Questionnaire; PCS = Pain Catastrophizing Scale; PDQ = Pain Disability Questionnaire; MEDD = morphine equivalent daily dose; ^&^numeric rating scale from 0 (no pain) to 10 (worst pain imaginable); ^∗^correlation is significant at the 0.05 level (2-tailed); ^∗∗^correlation is significant at the 0.01 level (2-tailed).

**Table 4:** Examination of changes in pre- and posttreatment measures.

	*t*	df	Significance (2-tailed)
Usual pain^&^	1.77	33	0.09
Acceptable pain^&^	−4.87^∗^	31	0.00
PHQ9	4.47^∗^	33	0.00
PCS	4.75^∗^	29	0.00
PDQ	4.38^∗^	34	0.00
MEDD	1.88	33	0.07

PHQ9 = Patient Health Questionnaire; PCS = Pain Catastrophizing Scale; PDQ = Pain Disability Questionnaire; MEDD = morphine equivalent daily dose; ^&^numeric rating scale from 0 (no pain) to 10 (worst pain imaginable); ^∗^correlation is significant at the 0.05 level (2-tailed).

**Table 5:** Linear regression analysis predicting pre-post PDQ.

	Coefficients	*t*	Significance
ΔPHQ9	0.45	2.90	0.00
ΔAcceptable pain^&^	−0.03	−0.21	0.84
ΔPCS	0.18	1.11	0.28

^a^Standardized coefficients are shown; stepwise linear regression models with changes in pain disability (PDQ) as dependent variables and three pre-post predicting factors: depression (PHQ9), acceptable level of pain, and pain catastrophizing (PCS); PHQ9 = Patient Health Questionnaire; PCS = Pain Catastrophizing Scale; PDQ = Pain Disability Questionnaire; ^&^numeric rating scale from 0 (no pain) to 10 (worst pain imaginable).
